# Health-related quality of life, compliance and sustained adherence on a low-carbohydrate high-fat diet compared with a high-carbohydrate low-fat diet in people with type 2 diabetes

**DOI:** 10.1016/j.ajcnut.2026.101211

**Published:** 2026-01-27

**Authors:** Ellen Elise Petersen, Johanne Kragh Hansen, Nikolaj Torp, Eva Gram-Kampmann, Peter Andersen, Stine Johansen, Ida Falk Villesen, Katrine Tholstrup Bech, Katrine Holtz Thorhauge, Helle Lindholm Schnefeld, Charlotte Mary Bastida Gjøl, Ellen Lyngbeck Jensen, Sönke Detlefsen, Kurt Højlund, Maja Thiele, Mads Israelsen, Aleksander Krag, Camilla Dalby Hansen

**Affiliations:** 1 Department of Gastroenterology and Hepatology, Odense University Hospital, Odense, Denmark; 2 Department of Clinical Research, University of Southern Denmark, Odense, Denmark; 3 Steno Diabetes Center Odense, Odense University Hospital, Odense, Denmark; 4 Department of Pathology, Odense University Hospital, Odense, Denmark

**Keywords:** dietary intervention, low-carbohydrate high-fat (LCHF), high-carbohydrate low-fat (HCLF), health-related quality of life (HRQoL), sustained adherence, metabolic dysfunction-associated steatotic liver disease (MASLD), type 2 diabetes mellitus (T2D)

## Abstract

**Background:**

Health-related quality of life (HRQoL) is a central aspect of overall health and a crucial factor in dietary interventions, as it may determine both dietary compliance and sustained adherence.

**Objectives:**

We assessed the effect on HRQoL between: *1*) the low-carbohydrate high-fat (LCHF) diet and *2*) the high-carbohydrate low-fat (HCLF) diet and evaluated the impact on dietary compliance and sustained adherence.

**Methods:**

This is a prespecified secondary analysis from a randomized controlled trial in people with type 2 diabetes. Participants were randomly assigned 2:1 to follow either LCHF or HCLF for 6 mo with a postintervention visit 9 mo after inclusion. Liver biopsies were performed at baseline and after 6 mo, the Diabetes-39 HRQoL questionnaire, standard clinical and compliance assessments were conducted at baseline, 3 mo, 6 mo, and 9 mo (postintervention). Sustained adherence was assessed at the postintervention visit.

**Results:**

We randomly assigned 165 participants; 96 (58%) were female. At baseline, the median age was 56 [interquartile range (IQR) 50–63] y, mean body mass index was 33 + 7 kg/m^2^, total median HRQoL score was 88 (IQR, 70–111), mean hemoglobin A1c was 56+10 mmol/mol, and 141 (88%) had metabolic dysfunction-associated steatotic liver disease. After 6-mo intervention, HRQoL improved in both groups {LCHF: −14.5 [95% confidence interval (CI): −20.7, −8.36]; *P* < 0.001; HCLF; −13.7 (95% CI: −22.7, −4.6); *P* = 0.003} with no mean difference in change between groups (Δ) *P* = 0.855. Higher improvements in HRQoL were associated with a higher compliance with the diets (Spearman’s rho; −0.183; *P* = 0.0378) and increased the likelihood of sustained adherence to the LCHF diet.

**Conclusions:**

HRQoL improved in both dietary intervention groups with no difference between groups. Dietary compliance was associated with improved HRQoL and may play a role in sustained adherence to the LCHF diet.

This trial was registered at clinicaltrials.gov as NCT03068078.

## Introduction

It is well established that long-term success of any dietary approach depends on the ability to comply with the regimen and to maintain adherence over time [1]. Adherence to dietary interventions poses one of the greatest unsolved challenges in nutritional therapy [2]. Sustained dietary adherence is influenced by multiple factors, including physical and mental well-being as well as practical feasibility [3,4]. These barriers have contributed to high dropout rates in dietary studies, limiting the real impact of otherwise effective interventions [5].

Although the low-carbohydrate high-fat (LCHF) diet has demonstrated positive effects on clinical outcomes such as hemoglobin A1c (HbA1c) and weight [[6], [7], [8]], evidence regarding its impact on quality of life and adherence in individuals with type 2 diabetes (T2D) remains limited. A study by Guldbrand et al. [9] suggested potential benefits of a low-carbohydrate diet on health-related quality of life (HRQoL) in patients with T2D; however, their sample size was small, and the dietary intervention did not align with current definitions of a low-carbohydrate diet [10].

Moreover, individuals respond differently to dietary interventions, both in terms of clinical outcomes [11] and HRQoL [12]. Although some experience great benefits in energy levels, mood, and overall well-being, others do not experience the same [12]. These differences in HRQoL may substantially influence dietary compliance and sustained adherence. Identifying those who improve their HRQoL, and how this aligns with clinical responses and adherence patterns, may provide valuable insights for developing more personalized and sustainable dietary strategies in the future.

In this prespecified secondary analysis of a 6-mo dietary randomized controlled trial in individuals with T2D [6], we investigated the impact of the LCHF diet and the high-carbohydrate low-fat (HCLF) diet on HRQoL, dietary compliance, and sustained adherence.

## Methods

We report a prespecified secondary analysis of the results from the project; A reduced-carbohydrate diet high in monounsaturated fats in type 2 diabetes: a six-month study of changes in metabolism, liver and cardiovascular function (REDUCTION). Full trial details have been reported previously [6]. REDUCTION was a single center, 6-mo randomized controlled dietary trial comparing a LCHF diet with a HCLF diet in individuals with T2D. Participants were recruited from the 29 November, 2016 and continued until 30 June, 2020. The final follow-up visit was conducted on the 4 June, 2021. Assessments were conducted at baseline, 3 mo, 6 mo [end of intervention (EOI)], and 9 mo (postintervention). Primary endpoints were the change in HbA1c, serum cholesterol, blood glucose, and metabolic markers. The trial was conducted at the Departments of Hepatology and Endocrinology, Odense University Hospital, Denmark. It was approved by the Ethics Committee of the Region of Southern Denmark (S-20150217) and the Danish Data Protection Agency (16/21793) and conducted in accordance with the Declaration of Helsinki. All participants provided written informed consent. The trial is registered at clinicaltrials.gov (NCT03068078**;** https://clinicaltrials.gov/study/NCT03068078).

### Participants and interventions

Participants were recruited via social media advertisements, local diabetes associations, and at outpatient clinics at Odense University Hospital. Key inclusion criteria were a diagnosis of T2D as defined by the criteria of the American Diabetes Association [13], whereas exclusion criteria included weight loss of >10 kg within the previous 3 mo, recent changes in medication, excessive alcohol intake defined by the Danish Health Authority as >7 units/wk for women (12 g/d) and 14 units/wk for men (24 g/d), or prior adherence to a low-carbohydrate diet. Participants were randomly assigned in a 2:1 ratio to either an LCHF or HCLF diet. Both diets were calorie-unrestricted, promoted healthy food choices, and emphasized reduced sugar intake. The LCHF diet was composed of ≤20% of daily energy (E%) from carbohydrates, 50E% to 60E% from fats, and 25 E% to 30E% from protein. In contrast, the HCLF diet provided 50 E% to 60E% carbohydrates, 20 E% to 30E% fats, and 20 E% to 25E% protein. Participants were able to contact site staff at any time with questions about the diets and permitted foods. Dietary compliance was assessed using a validated online food diary (MADLOG) [14] based on self-reported dietary intake, in collaboration with a clinical dietitian. Compliance was evaluated at 3 and 6 mo and categorized as <60%, 70%, 80%, 90%, and 100% compliance. The score reflected the proportion of time participants adhered to the assigned dietary regimen, informed by entries recorded in the food diary. At the EOI, participants were encouraged to follow their own dietary preferences. Sustained adherence was assessed at the postintervention visit through a structured interview. Participants were categorized as adherent if they reported following their assigned diet “most of the time,” which we considered reflective of predominant adherence in real-life settings.

### Diabetes-39 questionnaire

To assess HRQoL, we used the Diabetes-39 (D-39), a validated self-report instrument designed for individuals with type 1 and T2D [15]. The questionnaire comprises 39 items covering 5 dimensions: *Energy and Mobility* (15 items), *Sexual Function* (3 items), *Social Burden* (5 items), *Diabetes Control* (12 items), and *Anxiety and Worry* (4 items). Each item used a 1 to 7 scale, with 1 indicating the least impact on HRQoL and 7 the most severe impact on HRQoL.

Additionally, the questionnaire included 2 standalone items, which were also evaluated:

1. General quality of life: “Please mark the box that best describes your assessment of your overall quality of life.” Responses ranged from 1 (lowest quality) to 7 (highest quality).
2. Perceived diabetes severity: “Please mark the box that indicates how severe you consider your diabetes to be.” Responses followed the same 1 to 7 scale, with 1 indicating least and 7 most severe.

The D-39 questionnaire was originally developed to assess HRQoL in people with diabetes [15]. Participants completed the questionnaire at baseline, 3 mo, 6 mo, and 9 mo. The questionnaires were hosted in REDCap [16], and completed independently by participants on a tablet at the end of each visit, after all other assessments were finalized. A clinician was present in the room but did not monitor participants’ responses, though the participants were able to ask clarifying questions.

### Clinical assessments

Percutaneous liver biopsies were performed at baseline and at 6 mo using the Menghini technique. Biopsy specimens were immediately fixed in formalin and embedded in paraffin. All samples were evaluated by the same experienced hepatopathologist, who was blinded to group allocation and clinical data, using the Nonalcoholic Steatohepatitis Clinical Research Network histological scoring system [17]. Fasting blood samples were collected at each study visit and analyzed for standard metabolic parameters. Insulin resistance, assessed by HOMA-IR, was measured at baseline, 6 mo, and at postintervention (9 mo). Body weight was measured using the same calibrated scale across all visits.

### Statistical analysis

The trial was powered to detect a 0.7% difference in HbA1c (SD 13.0) with 80% power and a 5% significance level, requiring 135 participants (2:1 allocation); an additional 50 were enrolled to ensure adequate liver histological representation as prespecified in the protocol. Descriptive data are presented as mean ± SD for normally distributed variables and as median with IQR for non-normally distributed variables. Normality of the raw data was assessed using histograms. To assess changes in HRQoL, scores from all 41 questions were summed into a single total score ranging from 41 to 287. Changes in HRQoL from baseline to 9 mo were evaluated using mixed-effects models. Linear mixed-effects models were applied for continuous outcomes (total D-39 score and each of the 5 D-39 dimensions), and ordinal mixed-effects models for ordinal outcomes (overall QOL and perceived diabetes severity). Each model included visit, diet group, and a visit-by-group interaction as fixed effects, with subject ID and visit modeled as random effects using an unstructured covariance matrix. Normality assumptions were assessed through visual inspection of model residuals. Sex differences in intervention effects were evaluated by including a sex-by-group interaction term in the mixed-effects models. To illustrate the distribution of individual HRQoL responses, we categorized participants according to whether they improved or declined by >10%. This approach was not intended to define clinical significance but rather to provide a visual representation of response patterns within each group. To enable more stable comparison of adherence patterns, the original 5 narrowly spaced percentage categories (<60%, 70%, 80%, 90%, and 100%) were consolidated into 3 predefined groups: high (≥90%), medium (80%–89%), and low (<80%). Associations between compliance levels and changes in HRQoL, HbA1c, weight, and NAFLD activity score (NAS) were assessed using Spearman’s rank correlation. Group differences were tested using chi-square tests. To minimize unnecessary data loss, missing values were imputed using the most recent available response from the participant. This procedure affected <0.5% of all questionnaire items (92/22,960 responses) and reduced the proportion of missing items to 0.04% (9/22,960 responses). The remaining missing items resulted in exclusion of the respective questionnaires from the per-protocol analyses. All statistical analyses were performed using STATA (StataCorp) version 18. A 2-sided *P* value < 0.05 was considered statistically significant.

## Results

Between November 2016 and June 2020, we screened 777 individuals with T2D; 185 were eligible and provided consent, and 165 proceeded to the baseline visit and were included in the intention-to-treat analysis as previously reported [6]. Of these, 134 (81%) completed the trial; 92 (84%) in the LCHF group and 42 (76%) in the HCLF group. Postintervention data (3 mo after the EOI) were available for 122 participants (74%) (Supplementary Figure 1).

### Baseline characteristics

Baseline characteristics are reported in Table 1. Participants had a median age of 56 y (IQR: 50–63), 96 (58%) were female, the mean BMI was 33 kg/m^2^ (SD 7), and the mean HbA1c was 56 mmol/mol (SD 10). A total of 88% had MASLD, and the median NAFLD activity score was 3 (IQR: 2–4). Duration of T2D was evenly distributed between groups: 38% were diagnosed <5 y ago, 31% 5 to 10 y ago, and 31% >10 y ago.

**TABLE 1:** Baseline characteristics of the study population

Variable	Overall sample (*n =* 20,165)	LCHF (*n =* 20,110)	HCLF (*n =*2055)
Age (y) (IQR)	56 (50–63)	56 (60–62)	56 (50–63)
Female, *n* (%)	96 (58)	62 (56)	34 (62)
Diabetes duration			
<5 y *n*(%)	71 (38)	42 (38)	23 (42)
**5**–10 y *n* (%)	57 (31)	35 (32)	18 (33)
**>**10 y *n* (%)	57 (31)	33 (30)	14 (25)
BMI (kg/m^2^) (SD)	33 (7)	33 (7)	35 (7)
Metabolic syndrome *n* (%)	150 (91)	101 (92)	49 (89)
Hypertension *n* (%)	106 (65)	78 (67)	32 (59)
Low HDL cholesterol *n* (%)	107 (65)	73 (66)	34 (62)
BMI >25 *n* (%)	151 (92)	100 (91)	51 (93)
High waist circumference/central obesity *n* (%)	165 (100)	103 (94)	51 (93)
Hypertriglyceridemia *n* (%)	71 (43)	46 (42)	25 (45)
Weight kg (SD)	98 (22)	98 (23)	100 (22)
Liver histology			
Hepatic fibrosis, F0/F1/F2/F3/F4, *n*	18/94/43/5/1	14/64/27/2/1	4/3016/3/0
Hepatocellular steatosis, S0/S1/S2/S3, *n*	20/76/51/14	15/55/33/5	5/21/18/9
Hepatocellular inflammation, 0/1/2/3, *n*	24/109/27/1	21/68/18/1	3/41/9
Hepatocellular ballooning, 0/1/2, *n*	121/34/6	82/23/6	39/11/3
NAS (IQR)	3 (2–4)	2 (2–3)	3 (2–4)
Biochemistry			
HbA1c mmol/L (SD)	56 (10)	55 (10)	58 (10)
HOMA-IR (IQR)	6.3 (4.0–9.4)	6.0 (4.0–9.4)	6.9 (4.3–9.4)
LDL cholesterol mmol/L (SD)	2.2 (0.8)	2.2 (0.8)	2.3 (0.9)
HDL cholesterol mmol/L (SD)	1.2 (0.3)	1.2 (0.3)	1.1 (0.3)
Total cholesterol mmol/L (SD)	4.2 (0.9)	4.1 (0.9)	4.2 (1.1)

All 165 participants are included in these baseline characteristics. Normally distributed variables are presented as mean ± SD; non-normally distributed variables as median (IQR).

Abbreviations: HbA1C, hemoglobin A1c; HCLF, high-carbohydrate low-fat; LCHF, low-carbohydrate high-fat; NAS, NAFLD activity score.

### Clinical changes and adverse events between LCHF and HCLF

As previously reported [6], clinical changes by group can be found in Supplementary Table 1. From baseline to EOI, participants in the LCHF group had a mean change (Δ) in HbA1c of −9.5 mmol/mol compared with −3.5 mmol/mol in the HCLF group [ΔMD −6.05 mmol/mol (95% CI: −9.2, −3.0); *P* < 0.001]. Participants in the LCHF group lost 5.5 kg compared with 1.7 kg in the HCLF group [ΔMD −3.8 kg (95% CI: −6.2, −1.4); *P* = 0.003]. Regarding improvement in NAFLD activity score with ≥2 points, 17% improved in LCHF and 13% improved in HCLF. Adverse events were more frequently reported in the LCHF group, primarily related to gastrointestinal symptoms, which were most pronounced during the first 3 mo of the intervention (Supplementary Table 2).

### HRQoL between LCHF and HCLF

**FIGURE 1:**
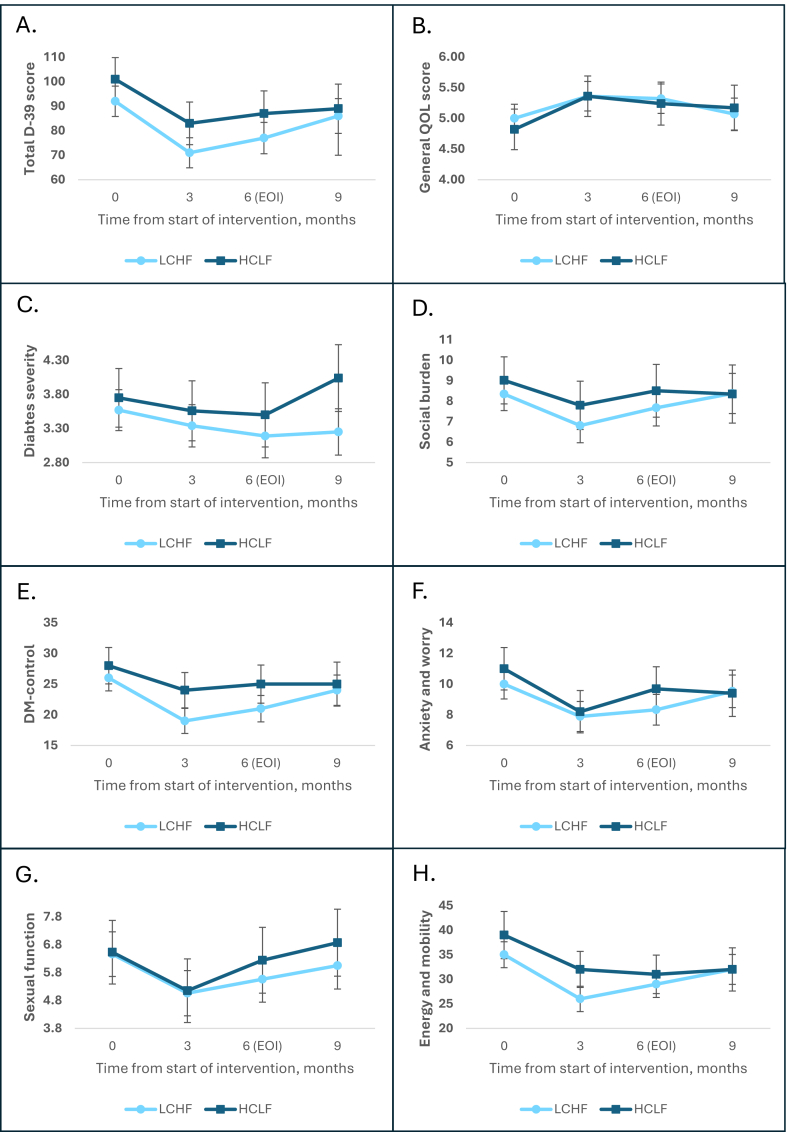
Changes in D-39 questionnaire over time between LCHF and HCLF. Analyses include all 165 participants and are based on mixed-effects models with visit, diet group, and their interaction as fixed effects, and participant as a random effect with an unstructured covariance structure. Normality was assessed visually. Values are presented as mean with 95% CIs. (A) Mean of all 39 D-39 items combined over time. Higher scores indicate greater overall health-related quality of life burden. (B) Single-item measure of self-rated overall quality of life. Responses ranged from 1 (lowest quality) to 7 (highest quality). Higher scores indicate better overall quality of life. (C) Single-item measure of self-rated diabetes severity. Responses ranged from 1 (least severe) to 7 (most severe). Higher scores indicate greater perceived disease burden. (D) Mean of the combined D-39 Social Burden domain scores over time. Higher scores indicate greater social burden. (E) Mean of the combined D-39 Diabetes Control domain scores over time. Higher scores indicate greater diabetes-related burden. (F) Mean of the combined D-39 Anxiety and Worry domain scores over time. Higher scores indicate greater anxiety-related burden. (G) Mean of the combined D-39 Sexual-Function domain scores over time. Higher scores indicate greater sexual-function burden. (H) Mean of the combined D-39 Energy and Mobility domain scores over time. Higher scores indicate greater energy- and mobility-related burden. CIs, confidence intervals; D-39, Diabetes-39 Questionnaire; LCHF, low-carbohydrate high-fat; HCLF, high-carbohydrate low-fat; HRQoL, health-related quality of life; T2D, type 2 diabetes mellitus.

From baseline to EOI, the combined overall HRQoL improved in both diets [LCHF: −14.5 (95% CI: −20.7, −8.3); *P* < 0.001; HCLF: −13.7 (95% CI: −22.7, −4.6); *P* = 0.003] with no significant difference between interventions groups [ΔMD: 0.84 (95% CI: −10.1, 11.8); *P* = 0.855] (Figure 1A). This corresponded to an improvement across both groups in the single-item general QOL (Figure 1B). When focusing on the 5 D-39 dimensions, no significant differences were observed between groups (Figure 1D–H). Across most dimensions, the greatest improvements occurred from baseline to 3 mo, followed by a partial return toward baseline by the EOI. At the postintervention visit, HRQoL had largely returned toward baseline levels. Perceived T2D severity worsened in the HCLF group after intervention compared with the LCHF group (Figure 1C). The distribution of HRQoL changes is shown in Supplementary Figure 2.

### Compliance to the dietary interventions

**FIGURE 2:**
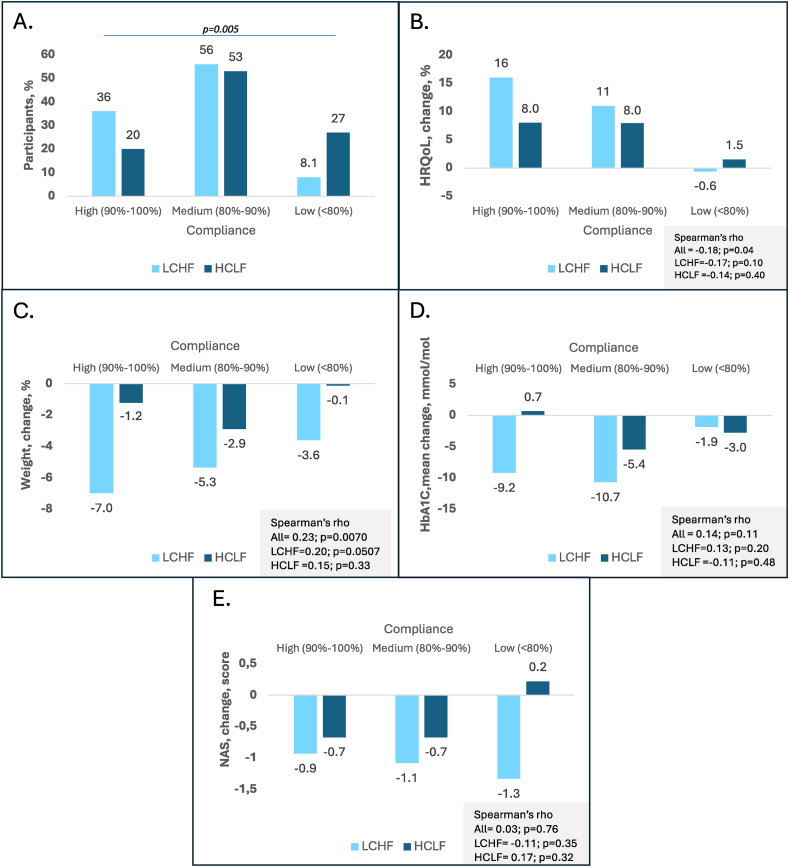
Compliance between groups and correlations with clinical parameters. This per-protocol analysis includes 135 participants. A Chi-square test was used to compare compliance levels between groups in (A), and Spearman’s rank correlation was applied to examine the associations between compliance and clinical outcomes shown in (B–E). (A) Distribution of participants by compliance level (high: 90%–100%, medium: 80%–90%, low: <80%). (B) Correlation between HRQoL and compliance levels between groups. (C) Correlation between mean weight change and compliance levels between groups. (D) Correlation between mean change in HbA1c and compliance levels between groups. (E) Correlation between mean change in NAS and compliance levels between groups. LCHF, low-carbohydrate high-fat; HbA1c, hemoglobin A1c; HCLF, high-carbohydrate low-fat; HRQoL, health-related quality of life; NAS, NAFLD activity score.

Throughout the 6-mo intervention, 46 participants (31%) had a high compliance, 81 (55%) had a medium compliance, and 21 (14%) had a low compliance across both intervention groups. Compliance was significantly higher on the LCHF diet compared with the HCLF diet (*P* = 0.005) (Figure 2A). Across both diets, participants with higher compliance demonstrated the greatest improvements in HRQoL (Spearman’s rho; −0.183; *P* = 0.038), whereas those with low compliance had minimal or no improvements (Figure 2B). Higher compliance was significantly associated with greater weight loss across both diets (*P =* 0.007), primarily driven by participants in the LCHF group (Figure 2C): participants with high compliance on the LCHF diet had a weight loss of −7.0% compared with –1.2% in the HCLF diet (Figure 2C). Among participants in the LCHF group, those with high and medium compliance achieved the largest reductions in HbA1c (−9.2 mmol/mol and −10.7 mmol/mol, respectively). In the HCLF group, HbA1c improved most among participants with medium compliance (−5.4 mmol/mol) (Figure 2D). Liver histology improved across all compliance levels in the LCHF group, whereas only participants with high and medium compliance in the HCLF group showed improvements in NAFLD activity score (Figure 2E).

### Sustained adherence to dietary interventions

**FIGURE 3:**
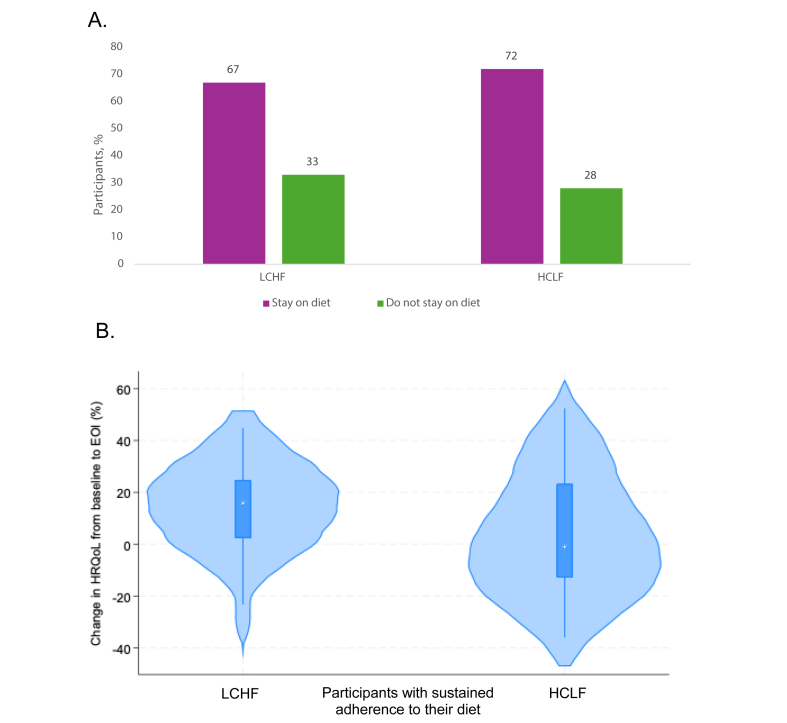
Sustained dietary adherence and associated changes in HRQoL. This analysis included 122 participants with available postintervention data. (A) Proportion of participants in each diet group (LCHF and HCLF) who reported continuing or discontinuing their assigned diet 3 mo after the end of intervention. (B) Distribution of percentage change in overall HRQoL from baseline to end of intervention among participants who reported sustained adherence to their assigned diet. Violin plots display the distribution, with the box representing the IQR, the horizontal line the median, and the white dot the mean value. EOI, end of intervention; LCHF, low-carbohydrate high-fat; HCLF, high-carbohydrate low-fat; HRQoL, health-related quality of life.

At the postintervention visit, 79 (69%) continued to adhere to their assigned diet: 51 (67%) in the LCHF group and 28 (72%) in the HCLF group (Figure 3A). Baseline characteristics of participants with sustained adherence are shown in Supplementary Table 3, with no significant differences compared with those who did not adhere. In the LCHF group, a greater proportion of participants who improved HRQoL also sustained adherence (Figure 3B).

### Sex differences in clinical responses, compliance, and adherence

Clinical responses differed by sex. Females on the LCHF diet showed greater improvements in liver histology compared with those on the HCLF diet, with significant reductions in NAS (*P =* 0.032), mainly driven by improvements in steatosis (*P =* 0.020) (Supplementary Table 4). No significant between-group differences were seen in males. Inflammation also decreased more in females on the LCHF diet, although this did not reach statistical significance (Supplementary Table 4). Compliance patterns also varied by sex: females had higher compliance on LCHF than HCLF (*P =* 0.027), whereas no significant difference was observed among males. Sustained adherence did not differ significantly by sex in either diet group (Supplementary Figure 3).

## Discussion

We found that HRQoL improved in both the LCHF and HCLF groups, with no significant differences between them across the 5 dimensions or the overall HRQoL score. Participants who experienced improvements in HRQoL during the intervention were more likely to demonstrate high dietary compliance, and in the LCHF group, such improvements were particularly associated with sustained adherence.

Given the superior clinical outcomes in the LCHF group, including greater weight loss and reductions in HbA1c, the absence of a between-group difference in HRQoL was unexpected. Previous findings, such as those from the study “Effectiveness of the Optifast Program Compared with a Reduced-energy Food-Based Diet Plan on Body Weight” by Dainelli et al. [18], have shown a clear association between weight loss and improved HRQoL in individuals with obesity. This discrepancy suggests that other factors might have influenced HRQoL, such as adverse events, which were more prevalent in the LCHF group. However, our observations are consistent with findings from Guldbrand et al. [9] who reported no significant differences in HRQoL between low-carb and low-fat diets over 6 mo in individuals with T2D, despite a clinically meaningful weight loss. One potential explanation is that changes in HRQoL may reflect not only physiological improvements but also study context, participant expectations, and structured support. Indeed, studies with a no-treatment control group suggest that participation alone can substantially influence HRQoL. In a 12-wk trial by Sindler et al. [19] involving people with obesity, even the control group with no active intervention showed marked improvements in psychological well-being. This suggests that being monitored and supported in a structured research setting may positively influence participants’ perceived health and quality of life. These effects are echoed in findings by Dudekula et al. [20], who showed that frequent contact with healthcare professionals was strongly associated with clinical responses in individuals with steatotic liver disease undergoing weight loss treatment. Together, these data highlight the importance of the care environment and participant engagement as critical drivers of perceived well-being, independent of the specific intervention.

A particularly notable finding was that participants who improved HRQoL also demonstrated higher compliance with the dietary interventions. This supports the hypothesis that individuals who feel better on a given diet are more likely to comply with it. However, the direction of this association remains uncertain. It is unclear whether higher HRQoL results from better compliance or whether improved HRQoL facilitates greater compliance.

Furthermore, more individuals on the LCHF diet with sustained dietary adherence experienced an improvement in HRQoL. This highlights the importance of integrating HRQoL into clinical decision-making, not only as an outcome but as a potential predictor of sustained behavioral change. Supporting individuals in selecting a dietary strategy that aligns with both their physiological response and sense of well-being may enhance adherence, which is widely regarded as a key driver of long-term clinical success. Interestingly, this association between HRQoL improvement and adherence was only observed in the LCHF group, reinforcing the growing consensus that dietary interventions should be personalized. Emerging evidence, including studies by Zeevi et al. [11], has demonstrated solely interindividual variability in metabolic responses to specific foods, suggesting that a “one-size-fits-all” approach is not optimal, also suggested by Zelber-Sagi et al. [21]. Despite these interesting observations, we were unable to identify baseline characteristics or model predictors that could reliably forecast who would benefit most from each dietary strategy. This remains a critical area for future research: developing tools to match individuals with the dietary approach most likely to promote both clinical improvement and sustained quality of life.

Our study has several strengths, including high clinical quality and detailed participant characterization. Notably, participants were recruited from the general population, which supports the generalizability of our findings to real-world settings. An additional advantage was that our dietary interventions were not calorie-restricted, which makes the conditions more reflective of real-world dietary practices.

However, the study also has important limitations. The study was powered for clinical endpoints rather than HRQoL, because we cannot exclude the possibility of a true difference in HRQoL that the study was underpowered to detect. Dietary compliance was based on self-reporting through a validated dietary diary; however, because we lacked dietary recordings at the postintervention follow-up, sustained adherence relied only on the participants judgment, making these data susceptible to recall bias and social desirability bias [22], where participants may report behaviors that they believe will be viewed favorably by study staff. This could lead to an overestimation of sustained adherence and perceived success. In addition, there was a risk of a Hawthorne effect, where participants modify their behavior simply because they are being observed. However, such bias would likely affect both groups equally and therefore may have limited impact on the observed between-group comparisons. Another limitation was that the study was conducted at a single center in Denmark, and the majority of participants were of White European descent. This limits the generalizability of our results to more ethnically diverse populations and other geographic and cultural regions. Finally, our postintervention period was limited to 3 mo, which constrains our ability to draw conclusions about the sustainability beyond this period.

Future research should aim to identify biomarkers that can help pinpoint individuals most likely to benefit from specific dietary interventions both with respect to HRQoL and clinical aspects. This could include more in-depth clinical assessments, such as genetic profiling or omics-based analyses, to better understand the physiological mechanisms behind individual responses. Such knowledge could ultimately improve the success rate of dietary interventions by enabling more targeted and personalized approaches. However, our findings also suggest that clinical response alone may not be sufficient. It may always be essential to consider the individual’s preferences and perceived well-being when selecting a dietary strategy, as this could play a critical role in sustained adherence and overall success.

In conclusion, both groups experienced improvements in HRQoL, but no significant differences were observed between the dietary interventions after 6 mo. Instead, HRQoL was closely associated with compliance in both groups and with sustained adherence in the LCHF group specifically. These findings suggest that improvements in quality of life are more strongly associated with adherence to a chosen diet than with the specific type of dietary intervention.

## Author contributions

The authors’ responsibilities were as follows – EG-K, KH, CDH, AK: conception and design of the study; EGK, NT, SJ, CDH, JKH, SD: acquisition of data; EEP, PA, CDH: analysis and interpretation of data; EEP, CDH: drafting of the manuscript; KTB, HLS, IFV, MT, MI, CMBG, ELJ, PA: critical revision for important intellectual content and technical/logistical support; and all authors: contributed to the interpretation of the data, had responsibility for the final content, and approved the final manuscript.

## Data availability

Deidentified data and protocol are available on request at camilla.dalby.hansen@rsyd.dk. Danish Data Protection approval is required, and usage is subject to restrictions, including the condition that data may only be processed for statistical and scientific research purpose.

## Declaration of generative AI and AI-assisted technologies in the writing process

Generative AI was used to improve the clarity, grammar, and flow of the manuscript text. All content, including data interpretation and conclusions, was generated by the authors**.**

## Funding

The project received funding from the Novo Nordisk Foundation (NNF16OC0021038) (NNF20SA0069372), Region of Southern Denmark, University of Southern Denmark, Odense University Hospital, Danish Diabetes Academy, A.P. Møller Foundation, and Overlæge Johan Boserup og Lise Boserups Legat. The funders had no role in the design of the study, data collection, data analysis, interpretation of results, manuscript preparation, or the decision to submit the manuscript for publication.

## Conflict of interest

EEP, NT, EGK, PA, SJ, IFV, KTB, KT, HLS, CMBG, ELJ, SD, and KH have no conflict of interests. JKH: speakers fee from Norgine. MT: consultancy fee from Novo Nordisk, GSK, Boehringer Ingelheim, AstraZeneca; speakers fee from Echosens, Siemens Healthcare, Norgine, Takeda, Madrigal, Tillotts Pharma. Board member and co-founder of Evido. Board member in Alcohol & Society (non-governmental organization). MI: Received travel grants from Novo Nordisk. AK has served as speaker for Novo Nordisk, Norgine and Siemens and participated in advisory boards for GSK, Madrigal, Boehringer Ingelheim and Novo Nordisk, all outside the submitted work. Research support; Norgine, Siemens, Nordic Bioscience, Echosense. Board member and co-founder Evido CDH: speakers fee from Novo Nordisk.
