# Supplementary material for: Health-related quality of life, compliance and sustained adherence on a low-carbohydrate high-fat diet compared with a high-carbohydrate low-fat diet in people with type 2 diabetes
Source: Am J Clin Nutr. 2026 Jan 27;123(4):101211. doi: 10.1016/j.ajcnut.2026.101211 (PMC13084574; doi:10.1016/j.ajcnut.2026.101211)
Supplement: Multimedia component 1 [file mmc1.docx]

Supplementary Material

Health-related quality of life, compliance and sustained adherence on a Low-Carbohydrate High-Fat diet compared with a High-Carbohydrate Low-Fat diet in people with type 2 diabetes

Ellen Elise Petersen^1,2^, Johanne Kragh Hansen^1,2^, Nikolaj Torp^1,2^, Eva Gram-Kampmann^4^, Peter Andersen^1^, Stine Johansen^1,2^, Ida Falk Villesen^1,2^, Katrine Tholstrup Bech^1,2^, Katrine Holtz Thorhauge^1,2^, Helle Lindholm Schnefeld^1^, Charlotte Mary Bastida Gjøl^1^, Ellen Lyngbeck Jensen^1^, Sönke Detlefsen^2,3^, Kurt Højlund^4^, Maja Thiele^1,2^, Mads Israelsen^1^,^2^, Aleksander Krag^1,2*^, Camilla Dalby Hansen^1,2^

1) Department of Gastroenterology and Hepatology, Odense University Hospital, Denmark

2) Clinical Institute, University of Southern Denmark, Denmark

3) Department of Pathology, Odense University Hospital, Denmark

4) Steno Diabetes Center, Odense University Hospital

**Supplementary Figure 1. Flowchart of participants**

**
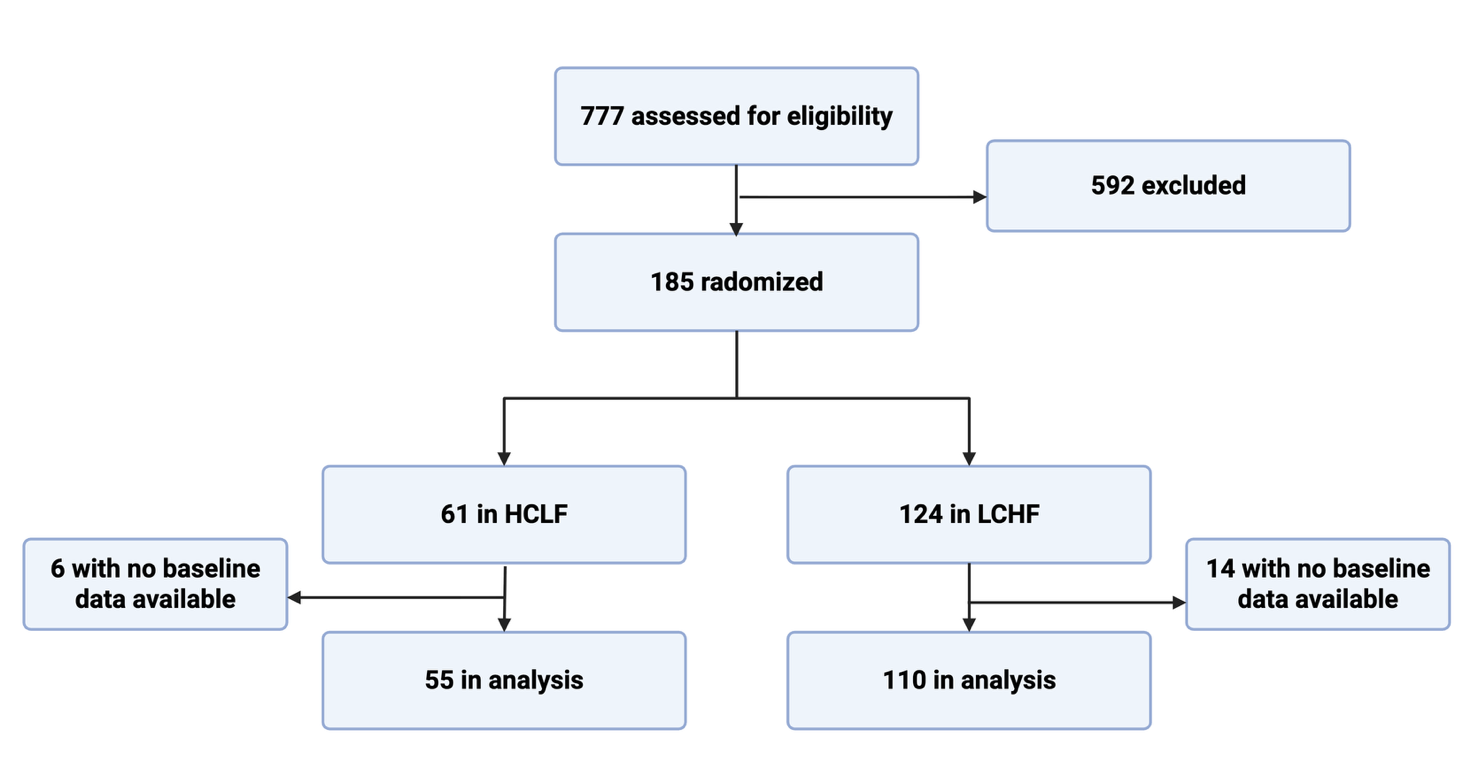
**

**Supplementary Table 1. Estimated Clinical changes from baseline to end-of-intervention for LCHF and HCLF**

| **Change** | **LCHF** | **HCLF** | **Mean difference in change** |
| --- | --- | --- | --- |
| HbA1c, mmol/mol | -9.52 (11.29, 7.75) | -3.47 (6.03, 0.91) | -6.05 (-9.15, -2.95) |
| Weight, kg | -5.5 (-6.8, -4.2 | -1.7 (-3.6, 0.2) | -3.8 (-6.2, -1.4) |
| NAS improvement of ≥ 2 points, % | 17% | 13% | - |

Mean changes (95% CI) in glycaemic control, body weight, and liver histology (NAS) from baseline to the end of intervention in participants randomised to a Low-Carbohydrate High-Fat (LCHF) or High-Carbohydrate Low-Fat (HCLF) diet. Between-group effects are presented as mean differences in change (95% CI). HbA1c = haemoglobin A1c; NAS = NAFLD activity score.

**Supplementary Figure 2. HRQoL change categories from baseline to EOI.**


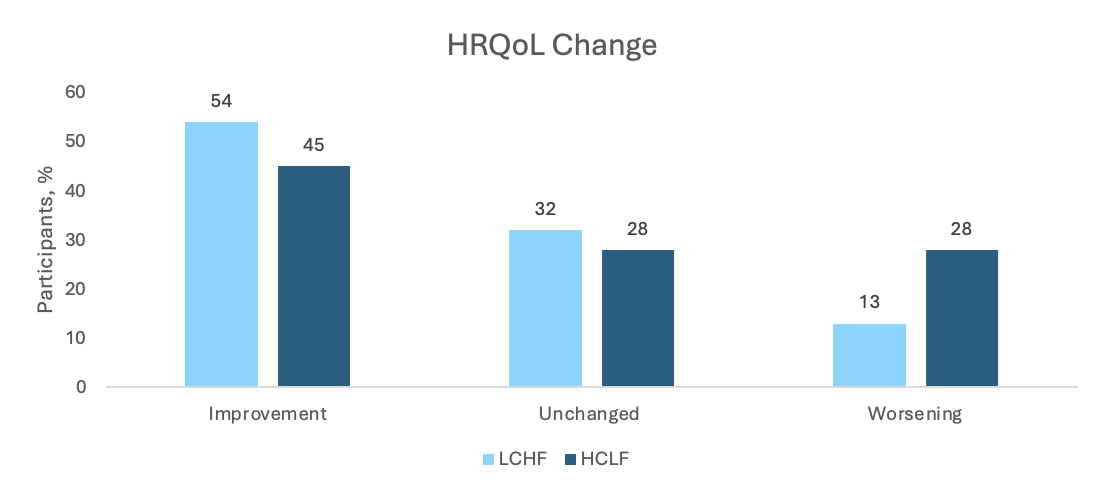


Participants were categorised as showing improvement (≥10% increase), no meaningful change (<10% increase and <10% decrease), or worsening (≥10% decrease) in HRQoL from baseline to end of intervention. These thresholds were applied for descriptive purposes to illustrate the distribution of change, rather than to define clinically validated cut-offs.
Abbreviations: LCHF = Low-carbohydrate High-Fat; HCLF = High-carbohydrate Low-fat; HRQoL = Health-related quality of life; EOI = End of intervention.

**Supplementary Table 2**

Baseline characteristics of the participants who adhere long-term and those who did not

Abbreviations: LCHF, Low-Carbohydrate High-Fat; HCLF, High Carb Low Fat; BMI, Body mass index; HbA1C, Hemoglobin A1c; TE, Transient Elastography; CAP, controlled attenuation parameter; HOMA-IR, Homeostatic Model Assessment for Insulin Resistance; LDL, Low density lipoprotein; HDL, High density lipoprotein. Normally distributed variables are presented as mean ± standard deviation (SD); non-normally distributed variables as median (interquartile range, IQR)
* Metabolic syndrome, low HDL, central obesity, hypertriglyceridemia defined per International Diabetes Federation (IDF) criteria .^22^
**Hypertension defined per European society of Cardiology (ESC).^23^
† F0: no fibrosis; F1: zone 3 or portal/periportal fibrosis only; F2: perisinusoidal and portal/periportal fibrosis; F3: bridging fibrosis; F4: cirrhosis.
†† S0: <5% steatosis; S1: 5%–33% steatosis; S2: 34%–66% steatosis; S3: >66% steatosis.
‡ 0: no foci per 200 field; 1: <2 foci; 2: 2–4 foci; 3: >4 foci.
‡‡ 0: none; 1: few; 2: many.

**Supplementary Table 3. Sex difference in response to the diets from Baseline to EOI.**

|  |  | **Estimated Mean Difference From Baseline (95% CI)** | | | |
| --- | --- | --- | --- | --- | --- |
| Change | Diet | Male | P-value | Female | P-value |
| Weight | LCHF | -4.04 (-5.24 to -2.84) | 0.03 | -6.56 (-7.67 to -5.45) | 0.000 |
|  | HCLF | -1.71 (-3.48 to 0.06) |  | -1.80 (-3.39 to -0.20) |  |
| HbA1c | LCHF | -10.56 (-13.66 to -7.44) | 0.000 | -8.68 (-10.70 to -6.66) | 0.004 |
|  | HCLF | -2.71 (-7.34 to 1.92) |  | -3.47 (-6.36 to -0.57) |  |
| NAS | LCHF | -1.63 (-2.69 to -0.58) | 0.558 | -2.27 (-3.25 to -1.29) | 0.032 |
|  | HCLF | -1.14 (-2.51 to 0.23) |  | -0.75 (-1.87 to 0.37) |  |
| Fibrosis | LCHF | -0.50 (-1.55 to 0.55) | 0.824 | 0.65 (-0.40 to 1.70) | 0.859 |
|  | HCLF | -0.71 (-2.24 to 0.82) |  | 0.49 (-0.99 to 1.96) |  |
| Ballooning | LCHF | -1.97 (-3.53 to -0.42) | 0.101 | -1.09 (-2.51 to 0.34) | 0.630 |
|  | HCLF | -0.06 (-1.74 to 1.61) |  | -1.64 (-3.48 to 0.20) |  |
| Inflammation | LCHF | -0.52 (-1.55 to 0.51) | 0.901 | -1.45 (-2.52 to -0.38) | 0.059 |
|  | HCLF | -0.41 (-1.90 to 1.08) |  | 0.19 (-1.11 to 1.48) |  |
| Steatosis | LCHF | -1.06 (-2.19 to 0.06) | 0.764 | -2.79 (-4.16 to -1.42) | 0.020 |
|  | HCLF | 1.35 (-2.91 to 0.22) |  | -0.74 (-2.07 to 0.59) |  |

Abbreviations: LCHF, Low-carbohydrate High-Fat; HCLF, High-carbohydrate Low-fat; HbA1C, Hemoglobin A1c; End of intervention.

**Supplementary Figure 3. Sex differences in dietary compliance and sustained adherence**

Compliance patterns and sustained adherence are presented separately for males and females to illustrate potential sex-specific behavioral responses to the dietary interventions. The association between sex and compliance was assessed using Spearman’s rho, and differences in long-term adherence were analyzed using Chi-square tests. **A.** Distribution of compliance levels (High: 90–100%, Medium: 80–90%, Low: <80%) by sex within each diet group (LCHF and HCLF).
**B.** Proportion of males and females who reported continued adherence to their assigned diet three months after end-of-intervention (“Stay” vs. “Do not stay”).

**Supplementary Table 4. Adverse events reported during the dietary intervention**

|  | **LCHF** | **HCLF** |
| --- | --- | --- |
| Mild Gastrointestinal symptoms |  |  |
| 2 weeks, n | 27 | 1 |
| 3 months, n | 23 | 4 |
| 6 months, n | 11 | 0 |
| Other mild symptoms |  |  |
| 2 weeks, n | 56 | 1 |
| 3 months, n | 4 | 1 |
| 6 months, n | 5 | 0 |

Frequency of mild gastrointestinal symptoms and other mild adverse events reported at 2 weeks, 3 months, and 6 months among participants following the Low-Carbohydrate High-Fat (LCHF) or High-Carbohydrate Low-Fat (HCLF) diet.
